# A Randomized Controlled Trial to Increase HIV Testing Demand Among Female Sex Workers in Kenya Through Announcing the Availability of HIV Self-testing Via Text Message

**DOI:** 10.1007/s10461-018-2248-5

**Published:** 2018-08-14

**Authors:** Elizabeth A. Kelvin, Gavin George, Eva Mwai, Samuel Kinyanjui, Matthew L. Romo, Jacob O. Odhiambo, Faith Oruko, Eston Nyaga, Kaymarlin Govender, Joanne E. Mantell

**Affiliations:** 1 Department of Epidemiology and Biostatistics, CUNY Graduate School of Public Health and Health Policy, City University of New York, 55 West 125th Street, New York, NY 10027 USA; 2 Institute for Implementation Science in Population Health, City University of New York, New York, NY USA; 3 Health Economics and HIV and AIDS Research Division, University of KwaZulu-Natal, Durban, South Africa; 4 North Star Alliance, Nairobi, Kenya; 5 Division of Gender, Sexuality and Health, Department of Psychiatry, HIV Center for Clinical and Behavioral Studies, New York State Psychiatric Institute & Columbia University, New York, NY USA

**Keywords:** HIV testing, HIV self-testing, Female sex workers, Kenya, Randomized controlled trial

## Abstract

We assessed whether informing female sex workers about the availability of HIV self-testing at clinics in Kenya using text messages would increase HIV testing rates. We selected a sample of 2196 female sex workers registered in an electronic health record system who were irregular HIV testers and randomized them to be sent a text message announcing the availability of (1) HIV self-test kits sent three times (intervention), (2) general HIV testing sent three times (enhanced standard of care [SOC]), or (3) general HIV testing sent one time (traditional SOC). Participants in the intervention arm were significantly more likely to test for HIV during 2-month follow-up compared to those in the enhanced SOC (OR 1.9, p = 0.001). There was no difference in HIV testing between those in the enhanced SOC and the traditional SOC arms. Announcing the availability of HIV self-testing via text message increased HIV testing among this high-risk group.

## Resumen

Evaluamos si informar a las trabajadoras sexuales femeninas acerca de la disponibilidad de la prueba autoadministrada del VIH en algunas clínicas en Kenia usando mensajes de texto aumentaría las tasas de prueba del VIH. Seleccionamos una muestra de 2196 trabajadoras sexuales femeninas registradas en un sistema electrónico de registros de salud que habían hecho la prueba del VIH irregularmente y las asignamos al azar para recibir mensajes de texto que anunciaba la disponibilidad de (1) botiquines de la prueba autoadministrada del VIH, enviado tres veces (intervención), (2) pruebas estándares del VIH, enviado tres veces (estándar de cuidado [EDC] aumentado), o (3) pruebas estándares del VIH, enviado una vez (EDC tradicional). Fue significativamente más probable que aquellos en el grupo de intervención hicieran la prueba del VIH durante el seguimiento de 2 meses en comparación con aquellos en el EDC aumentado (razón de momios = 1,9, p = 0,001). No hubo diferencias en hacer la prueba del VIH entre aquellos en el EDC aumentado y el EDC tradicional. Anunciar la disponibilidad de la prueba de VIH autoadministrada a través de mensajes de texto aumentó las tasas de prueba del VIH entre este grupo de alto riesgo.

## Introduction

Worldwide, HIV prevalence among sex workers is estimated to be as much as 12 times that of the general population [1]. In Kenya, where this study was conducted, the disparity is somewhat lower but still concerning, with HIV prevalence among sex workers about 30%, compared to 5.4% in the general population [2, 3]. The HIV testing rate among female sex workers in Kenya is higher than in the general population, but given their risk, it remains lower than ideal. In Kenya in 2013, an estimated 68.0% of female sex workers had tested for HIV in the past 12 months [2].

HIV self-testing may increase HIV testing rates among key populations such as sex workers. A number of studies have found that bringing HIV self-testing to potential users can increase HIV testing rates, including in studies in which pregnant women distributed self-test kits to their main partners in Kenya [4] and Uganda [5], studies in which peer educators provided self-test kits to female sex workers in Uganda [6] and Zambia [7], and a home-based (door-to-door) HIV testing study in Zambia which found that offering a self-test in addition to the standard provider-administered blood-based HIV test increased testing rates from 55.1 to 60.4% [5]. In a randomized controlled trial in 2015 among 305 truck drivers in Kenya, those offered oral HIV self-testing as a choice in addition to the standard provider-administered blood-based test when visiting a clinic had 2.8 times higher odds of accepting HIV testing compared to that of those only offered the standard HIV test (p = 0.002) [8, 9]. However, allowing those in the intervention arm to also access self-test kits from the clinics over 6-months follow-up had no impact on testing during that time (OR 1.0, p = 0.972) [8]. This suggests that although offering an HIV self-test kit to someone when they are with you (e.g., when they are already present in a clinic or by taking the self-test kit to them at home or work) may increase testing, awareness of the availability of HIV self-testing may not be sufficient to motivate people to come to a clinic to obtain the test kit. If this is the case, making HIV self-testing available through facilities such as clinics and pharmacies may fail to reach those who are not accessing healthcare services, which would likely limit the impact this new testing modality has on curbing the HIV epidemic.

We conducted a randomized controlled trial among female sex workers in Kenya who were irregular HIV testers to assess whether announcing the availability of HIV self-test kits in a clinic system in Kenya via text message would bring more female sex workers to the participating clinics for HIV testing compared to the standard of care text message reminder about HIV testing in general.

## Methods

### Setting

The study was conducted in eight roadside wellness clinics in Kenya run by the North Star Alliance. The North Star Alliance is an organization providing primary and secondary health services to hard-to-reach populations, including sex workers and truck drivers, through its 36 clinics located at major transit hubs across Africa. In 2015, the North Star Alliance provided services to 253,227 client-visits, of which 18% included HIV testing [10]. When a person visits any North Star Alliance clinic, his/her information is entered into the electronic health record system, including a mobile phone number if the client has one and is willing to share it.

### Standard of Care

At every client encounter in a North Star Alliance clinic, HIV testing is offered and the test used is a blood-based (finger-prick) provider-administered test. HIV testing by clients at North Star clinics is tracked in the electronic health record system and a few times a year a text message reminder is sent to those clients who do not have a record of having tested for HIV in the previous 3 months. The message for clients in East Africa reads “North Star Alliance East Africa would wish to kindly remind you to visit any of our roadside wellness centres for HIV testing. Your health, our priority.”

### Sample, Eligibility and Consent

For this study, we selected a sample of female sex workers registered in the North Star Alliance electronic health record system who: (1) had no indication that they were HIV-positive, (2) resided in Kenya, (3) had a valid mobile phone number listed, (4) had fewer than four HIV tests recorded in the system in the past 12 months (indicating that they were not following the recommendation to test every 3 months for four tests per year [11]), and (5) had not had an HIV test in the past 3 months.

Once the sample of eligible participants was selected and their data cleaned, the North Star Alliance sent the following passive consent text message twice, once in Kiswahili and once in English, a week apart.

North Star Alliance is evaluating our programs for their improvement using client information from our system. The information we use for this evaluation will not be linked to your name and you will not be contacted or have any expenses related to your inclusion. If you have questions about the use of your data, call [phone number of clinic where they had last been seen]. To have your data excluded, reply “NO” to this text.

After each consent message, any clients who contacted us indicating they wanted to opt out of having their data included were removed from the sample prior to randomization.

### Randomization and Intervention

The eligible individuals in our samples who did not communicate their desire to opt out of the evaluation were randomized to one of three study arms.

1. *Intervention* The intervention consisted of a text message informing participants that HIV self-test kits were available at all North Star Alliance clinics in Kenya. The message was sent three times, one week apart, first in Kiswahili, then in English and then again in Kiswahili, and read:
  You can now self-test at home or in the clinic for HIV using a new test kit available from all North Star Alliance clinics in Kenya. Your health, our priority.
2. *Enhanced Standard of Care (Enhanced SOC)* Those randomized to the enhanced SOC arm received the SOC message reminding clients to come to a clinic for HIV testing (described above under *Standard of Care*), sent three times, one week apart, first in Kiswahili, then in English and then again in Kiswahili.
3. *Traditional Standard of Care (Traditional SOC)* Those randomized to the traditional SOC arm received the SOC message one time sent simultaneously in both Kiswahili and English.

### Sample Size and Power

Our primary outcome of interest was the comparison of HIV testing rates over a 2-month period following the initial text message about HIV testing between the intervention and enhanced SOC arms. We calculated sample size assuming the enhanced SOC would achieve 48% testing rate (38–48% were expected to test after a text reminder based on past records in the electronic health record system), and found that in order to detect a risk ratio (RR) of 1.2 (odds ratio [OR] 1.4) at 80% power and 95% confidence level, we would need a sample of about 750 female sex workers in each study arm. Therefore, we set our target sample size to 750 in each of the two study arms of primary interest, the intervention and enhanced SOC, and determined the randomization ratio after selecting the eligible sample in order to achieve this goal, which ended-up being 1:1:0.93 for the intervention, enhanced SOC, and SOC arms respectively.

### HIV Testing Procedures

Study participants who presented at any North Star Alliance clinic in the SOC arms (enhanced or traditional) were offered only the standard provider-administered blood-based HIV test, which is offered to all North Star Alliance clinic attendees. Participants from the intervention arm who presented at a North Star Alliance clinic in Kenya were given a brief demonstration of the self-testing kit and then offered a choice among: (1) the standard provider-administered blood-based HIV test; (2) the self-administered oral HIV test for use in the clinic with provider supervision; or (3) the self-administered oral HIV test kit for home use. Study arm was identified by the clinic receptionist by looking-up the client’s mobile phone number on an Excel spreadsheet listing the numbers of those in the intervention arm. The counselor was informed when an intervention client came in so she would be given a demonstration of the self-test kit and then offered the testing choices. Those in all study arms who visited a North Star Alliance clinic outside of Kenya would be offered the SOC test only, as those clinics did not have self-test kits. In addition, if someone not in the intervention arm came to a Kenyan clinic and specifically requested a self-test kit, presumably having heard about it from someone in the intervention arm, they were given the self-test so as not to miss an HIV testing opportunity. The HIV testing procedures were as follows:

Those who accepted the standard provider-administered blood-based test underwent the standard pre- and post-test counseling and testing process.

Those who chose the self-test for supervised use in the clinic received the standard pre-test counseling and then were given an OraQuick HIV self-test kit [12] which has an insert with written (English and Kiswahili) and pictorial instructions included as part of the kit. An HTC counselor sat in a private room with the study participant while she used the HIV test (supervised self-administration) in order to answer any questions that arose during the test administration and offer correction if needed. Upon the availability of the HIV test results 20 min later, the client was given the option to view the results in private or with the counselor. After viewing the HIV test results, the client received the standard post-test counseling and any needed referrals. If the client chose to view the test results in private, she was encouraged to disclose the test results during post-test counseling, but the final decision whether or not to disclose was the client’s. If she did not disclose the results, the counselor was to provide the post-test counseling information for both scenarios (HIV-positive and HIV-negative test result), including information about accessing HIV care in the case of a positive test result.

Those who chose to take a self-test kit for use outside of the clinic (i.e., home use) were given pre-test counseling in the clinic and then instructed to use their test within 3 days and to call or send a text message after using the test to receive a call-back for post-test counseling and any necessary referrals. Participants who failed to contact the clinic staff within 3 days after obtaining a test kit were called to inquire about the use of the test kit and provided counseling and referrals if needed. Clients were also told that they could call or send a text at any time while self-testing should they have any questions or concerns. As with in-clinic self-testing, clients were encouraged to disclose their test results during post-test counseling, but whether or not they did so was the client’s choice and if she did not disclose, the counselor was to provide information about both HIV test outcome scenarios.

### Data Collection Methods

For this study, we relied on data from two sources: (1) the North Star Alliance electronic health record system which documented HIV testing and which test was used (provider-administered test or self-test), and (2) administrative data collected at the clinics in a password-protected Excel spreadsheet for tracking the number of self-test kits used to order resupplies when needed and for tracking time since a client took a self-test kit for home use to ascertain when to contact the client if they failed to call for post-test counseling.

The study procedures were approved by the City University of New York Institutional Review Board, the Kenya Medical Research Institute Ethics Committee, and the University of KwaZulu-Natal Biomedical Research Ethics Committee.

### Data Analysis

We described the sample in terms of the basic demographic characteristics (those available in the health record system) overall and by study arm and assessed the statistical significance of any differences by study arm using a Chi square test for categorical variables and a Kruskal–Wallis test for numeric variables. We then conducted logistic regression analysis to compare HIV testing during the 2-month follow-up period among clients in the intervention arm versus those in the enhanced SOC arm (primary comparison) as well as among those in the enhanced SOC versus those in the traditional SOC arms (secondary comparison) to look at the impact of the content of the text message (i.e., about self-testing kits or HIV testing in general) and of the number of text messages (3 vs. 1) on HIV testing, respectively. We also used logistic regression to look at differences in clinic contact for any reason (i.e., for HIV testing or some other service) between the groups to see if the text message brought more clients to the clinic even if some chose not to test. Finally, we looked at whether the differences in HIV testing by study arm were modified by HIV testing history (whether the client had an HIV test at a North Star Alliance clinic in the past year) and, for those in the intervention arm, we describe the HIV testing choices made.

We found some discrepancies between the electronic health record data and the clinic administrative records that were kept on self-testers in the number of people who self-tested for HIV in the intervention arm. Specifically, there were 38 female sex workers whose data in the electronic health record system did not indicate an HIV test but the clinics listed them as having self-tested. This might occur for a number of reasons, such as the counselor forgot to enter the data in the online health record system, or entered it but the internet connection was disrupted while the data were being sent to the server, or the data were entered after we downloaded the data for these analyses. The data were downloaded from the health record system 2 months after completing follow-up. Because of this discrepancy, we first analyzed the data including these 38 female sex workers as not having tested (as indicated in the electronic health records) because we did not have similar administrative data on HIV testing from the clinics for those in the SOC arms. Differential data cleaning could bias the results and incorrectly elevate the association between the intervention and HIV testing and we felt it best to err on the conservative side knowing that we might have bias toward the null. However, we also ran the analysis recoding those 38 female sex workers as having tested, as indicated in the administrative clinic records, to see if it changed our results substantively; while the strength of the effect increased as would be expected, the conclusions regarding the significance of the associations remained unchanged in all comparisons. In addition, after the study began, two participants in the intervention arm disclosed that they were HIV-positive to clinic staff when offered HIV testing. There was no indication in the electronic heath record system of their HIV-positive status. We included these two individuals in the intervention arm for analysis to maintain the randomization, despite the fact that it would bias our findings toward the null.

## Results

### Description of the Sample

**Fig. 1:**
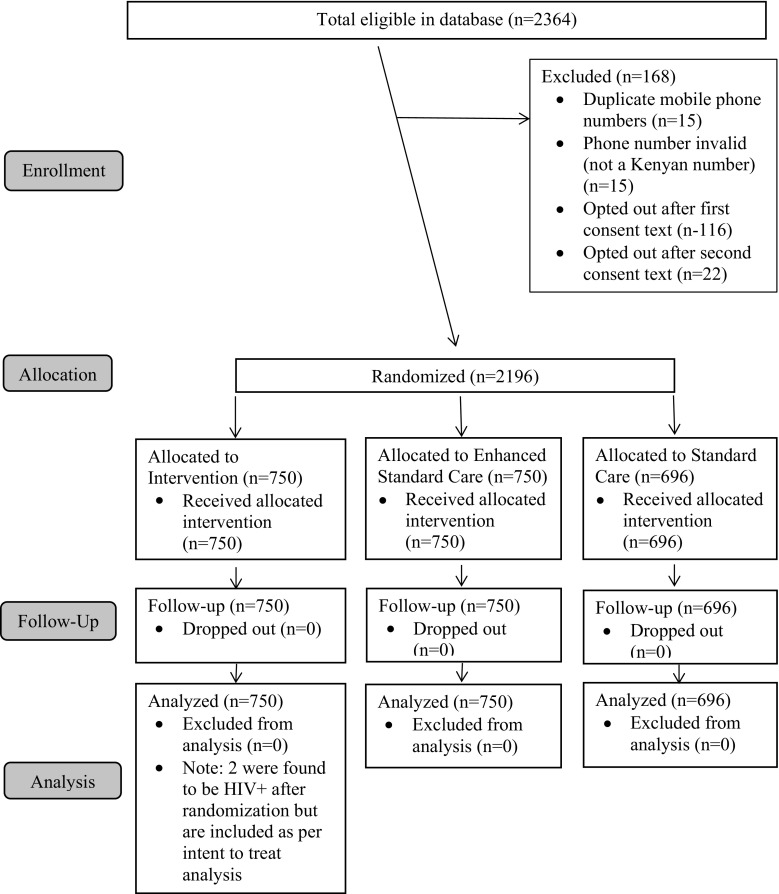
Flow of female sex workers participants (consort flowchart)

On February 13, 2017, we selected the sample of 2364 female sex worker clients from the electronic health record system who met the eligibility criteria. We deleted duplicate phone numbers from the sample, leaving us with a sample of 2349 female sex workers to whom we sent the first consent text message, after which 116 female sex workers contacted us to opt out and 15 phone numbers were returned as invalid. A week later we sent the second consent text message, after which an additional 22 female sex workers contacted us to opt out. On March 2, 2017, the remaining 2196 female sex workers were randomized to the intervention (n = 750), enhanced SOC (n = 750) or traditional SOC (n = 696) arms and the first study text messages were sent according to their study arm. The text messages were sent two more times, 1 week apart for those in the intervention and enhanced SOC arms (Fig. 1).

The mean age of the female sex workers was 28.6 years and only a few were married or living with a partner (8.8%). Overall, 64.7% had not had an HIV test in the past year, and among those who had tested in the past year, the mean time since testing was 6.3 months. There were no significant differences in these characteristics by study arm (Table 1).

**Table 1:** Descriptive statistics for female sex worker sample overall and by study arm

	Total	Intervention	Enhanced SOC	Traditional SOC	p value
Total, n (row%)	2196 (100.0%)	750 (34.2%)	750 (34.2%)	696 (31.7%)	NA
Age					0.408^a^
Mean (SD)	28.6 (5.9)	28.3 (5.9)	28.7 (6.0)	28.6 (5.9)	
Median (range)	28.0 (18.0–61.0)	28.0 (18.0–61.0)	28.0 (18.0–52.0)	28.0 (18.0–53.0)	
Marital status, n (column %)					0.356
Married/cohabitating	176 (8.8%)	69 (9.9%)	52 (7.7%)	55 (8.8%)	
Unmarried (single, divorced/separated)	1818 (91.2%)	625 (90.1%)	620 (92.3%)	573 (91.2%)	
Tested in past year, n (column %)					0.862^b^
Yes	776 (35.3%)	270 (36.0%)	265 (35.3%)	241 (34.6%)	
No	1420 (64.7%)	470 (64.0%)	485 (64.7%)	455 (65.4%)	
Months since last test among those tested in past year					0.144^a^
Mean (SD)	6.3 (2.6)	6.6 (2.6)	6.2 (2.6)	6.3 (2.6)	
Median (range)	5.5 (3.0–12.0)	6.3 (3.0–12.0)	5.5 (3.0–12.0)	5.5 (3.0–12.0)	
North Star Alliance Clinic where last seen, n (column %)					0.160^b^
Burnt Forest, Kenya	58 (2.6%)	19 (2.5%)	22 (2.9%)	17 (2.4%)	
Emali, Kenya	267 (12.2%)	90 (12.0%)	86 (11.5%)	91 (13.1%)	
Jomvu, Kenya	364 (16.6%)	121 (16.1%)	121 (16.1%)	122 (17.5%)	
Maai Mahiu, Kenya	265 (12.1%)	79 (10.5%)	105 (14.0%)	81 (11.6%)	
Mlolongo, Kenya	245 (11.2%)	71 (9.5%)	85 (11.3%)	89 (12.8%)	
Mombasa, Kenya	103 (4.7%)	43 (5.7%)	38 (5.1%)	38 (5.1%)	
Namanga, Kenya	185 (8.4%)	63 (8.4%)	69 (9.2%)	53 (7.6%)	
Salgaa, Kenya	709 (32.3%)	264 (35.2%)	224 (29.9%)	221 (31.8%)	

^a^p-value from Kruskal–Wallis test

^b^p-value from Chi square test

### Logistic Regression Models Comparing Those in the Intervention Arm to Those in the Enhanced SOC Arm

Forty-six (6.1%) participants in the enhanced SOC arm and 81 (10.8%) in the intervention arm tested for HIV during the 2-month follow-up period. Those in the intervention arm had 1.9 times greater odds of HIV testing compared to those in the enhanced SOC arm, which was statistically significant (p = 0.001). If we include the additional 38 female sex workers who had an indication of HIV self-testing in the administrative clinic records but not in the electronic health record system, the odds ratio increases to 2.9 (p < 0.001). (Table 2) The difference by study arm in HIV testing was not modified by having tested in the past year (interaction p value = 0.851) (data not shown).

**Table 2:** Differences in HIV testing and in receiving any clinic services comparing intervention to the enhanced SOC arms

	Total, n (%)	Enhanced SOC arm, n (column %)	Intervention arm, n (column %)	OR (95% CI)	Chi Square p-value
Total	1500 (100.0%)	750 (50.0%)	750 (50.0%)	NA	NA
Tested for HIV (according to electronic health record system only)^a^					
Yes	127 (8.5%)	46 (6.1%)	81 (10.8%)	1.9 (1.3–2.7)	0.001
No	1373 (91.5%)	704 (93.9%)	669 (89.2%)		
Tested for HIV (including the 38 participants who had an indication of HIV testing in clinic records but not in the electronic health record system)					
Yes	165 (11.0%)	46 (6.1%)	119 (15.9%)	2.9 (2.0–4.1)	< 0.001
No	1335 (89.0%)	704 (93.3%)	631 (84.1%)		
Received any clinic services (according to electronic health record system only)^a^					
Yes	175 (11.7%)	70 (9.3%)	105 (14.0%)	1.6 (1.2–2.2)	0.005
No	1325 (88.3%)	680 (90.7%)	645 (86.0%)		
Received any clinic services (including the 38 participants who had an indication of HIV testing in clinic records but not in the electronic health record system)					
Yes	199 (13.3%)	70 (9.3%)	129 (17.2%)	2.0 (1.5–2.8)	< 0.001
No	1301. (86.7%)	680 (90.7%)	621 (82.8%)		

^a^38 clients were noted as having HIV tested in the clinic administrative records used for tracking test kits and posttest counselling but their test was not indicated in the electronic health record system

Seventy participants (9.3%) in the enhanced SOC arm and 105 (14.0%) in the intervention arm had some form of clinic contact or service during the 2-month follow-up. Those in the intervention arm had 1.6 times greater odds of clinic contact compared to those in the enhanced SOC arm, which was statistically significant (p = 0.005). If we include the additional 38 female sex workers who had an indication of HIV self-testing in the administrative clinic records but not in the electronic health record system as having had clinic contact, the odds ratio increases to 2.0 (p < 0.001) (Table 2).

### Logistic Regression Models Comparing Those in the Enhanced SOC Arm to those in the Traditional SOC Arm

Overall, 43 (6.2%) participants in the traditional SOC arm compared to 46 (6.1%) in the enhanced SOC arm tested for HIV over the 2-month follow-up period, giving an odds ratio of 1.0 (p = 0.972). There was also no difference in clinic contact between the two arms (10.1% in the traditional SOC arm, 9.3% in the enhanced SOC arm, OR 0.9, p = 0.642) (Table 3).

**Table 3:** Differences in HIV testing and in receiving any clinic services comparing the enhanced SOC to the traditional SOC

	Total, n (column %)	Traditional SOC arm, n (column %)	Enhanced SOC arm, n (column %)	OR (95% CI)	Chi Square p-value
Total	1446	750 (52.0%)	696 (48.0%)	NA	NA
Tested for HIV					
Yes	89 (6.2%)	43 (6.2%)	46 (6.1%)	1.0 (7.0–1.5)	0.972
No	1357 (93.8%)	653 (93.8%)	704 (93.9%)		
Received any clinic services					
Yes	140 (9.7%)	70 (10.1%)	70 (9.3%)	0.9 (0.7–1.3)	0.642
No	1306 (90.3%)	626 (89.9%)	680 (90.7%)		

### HIV Testing Choices Made Among Those in the Intervention Arm

Of the 119 female sex workers who tested in the intervention arm (including the 38 identified in the administrative clinic records only), 71 (59.7%) chose to self-test. One person took two HIV self-test kits from two different clinics, so the number of self-test kits used was 72. Of those, 52 (72.2%) self-tested in the clinic with supervision and 20 (27.8%) were taken for home use.

Of the 20 test kits taken for home use, in 5 (25.0%) cases participants called while testing with questions, in 3 (15.0%) cases participants called after testing for posttest counseling, and in 7 cases (35.0%) participants called both while testing with questions and after for counseling. Five (25.0%) female sex workers did not call at all, and the counselor had to call them for posttest counseling. It took one attempt to reach three of these participants who did not call, two attempts for one participant and five attempts for the remaining participant. Five female sex workers tested HIV-positive during the study, all of whom were in the intervention arm (data not shown).

## Discussion

HIV testing at baseline was very low in this sample of female sex workers, with only 35.3% having had an HIV test in the past year. This was in part by design as the goal of the study was to look at the impact of HIV self-testing among irregular testers and not following the recommendation of testing every 3 months was an eligibility criterion. Testing rates during study follow-up were also low, at about 6% in both the enhanced and traditional SOC arms, suggesting that we have not been able to reach this group with traditional HIV testing options. Advertising self-test kits increased the HIV testing rate to 10.6% for an OR of 1.9 when using the health record data alone and 2.9 times when including the clinic record data.

Our findings that offering HIV self-testing increases HIV testing uptake are similar to those found in other trials. The impact of HIV self-testing on testing rates has ranged from a RR of 1.1–2.1, depending on the study design and population [4–9]. Thus, the impact of offering HIV self-testing seems to be fairly consistent across population groups, distribution methods (e.g., bringing the test kits to people or allowing people to access test kits from a clinic), and geographic region within sub-Saharan Africa.

Our use of text messaging in this study to announce the availability of a new HIV testing option is an important, low-cost innovation which could be useful as countries roll out self-testing in various venues. HIV self-testing was initiated in Kenya in May 2017, about one month after our study ended, with plans to make it available at public and private clinics and in pharmacies for a cost of about $8 US [13]. Text messages could be an easy and cost-effective tool to alert the public about the availability of HIV self-test kits as they are rolled out in more and more locations. Our text message was fairly simple, due to the character limits set for text messages, but the Pharmaceutical Society of Kenya has a website where videos of the self-testing process are available along with instructions in multiple African languages, including Kiswahili [14]. With the increasing proliferation of smart phones in Kenya [15], the link could be sent to people to aid in communication and understanding about the self-test kits and self-testing process, which might lead to a further increase in self-test uptake over what we achieved with such a simple message.

Despite the success of our intervention, the percent testing even with the self-testing intervention remained alarmingly low (10.8% in the intervention arm). Clearly offering self-testing at North Star Alliance clinics is only a partial solution to the low HIV testing rates among some female sex workers; other mechanisms will also be needed to increase testing rates in this high-risk group. One possible barrier may be the distribution mechanism. Distributing test-kits through clinics may not reach those who are unable to access or feel uncomfortable going to clinics. Although everyone in our study had received services from the North Star Alliance clinic system at least once in the past, as indicated by their registration in the health record system, some may have gotten those services via outreach instead of visiting a clinic. Other studies have looked at alternate distribution mechanisms for getting self-test kits to people, including pregnant women bringing self-test kits to male partners [4, 5], door-to-door delivery to people at their homes [5], and distribution by peer educators [6, 7]. Comparison of various distribution methods, especially for high risk groups such as female sex workers, should be explored. The North Star Alliance does HIV-testing outreach at truck stops and this might be a mechanism to conveniently distribute self-test kits. Demand creation may also be needed to reach some North Star Alliance clients with HIV testing, as well as those who are not accessing care in North Star or other clinic systems,

Among those in the intervention arm who were offered HIV testing choices, there was a range in which test participants selected. While the majority chose to self-test (59.7%), the SOC test was chosen by a fair number of participants. Furthermore, of those who opted to self-test, about two-thirds chose to self-test in the clinic with supervision while the others chose to take a test kit for home use. This suggests that people vary in their preferences around HIV testing and offering choices may be key to maximizing HIV-testing rates as it increases the chance that an acceptable option will be available. The self-testing study among truck drivers in Kenya found similarly varied choices [8, 9, 16], as did the Zambian study in which home-based HIV testing choices, including self-testing with and without supervision, were offered door-to-door [5]. The fact that a sizeable proportion of participants in all these studies chose to self-test in the clinic with supervision could be because some wanted guidance for their first time self-testing and going forward they would take a test kit to use at home. On the other hand, it could also be that some really preferred an oral HIV test over a blood test and were indifferent about who administered the test. A discrete choice experiment among truck drivers suggested that some have strong preferences regarding blood versus oral tests and the form of counseling (in-person versus over the phone) and these differed by HIV testing history, but preferences regarding who administers the HIV test and the testing location were not strong [8, 17]. Another study that made both oral and blood-based self-testing available to men who have sex with men in South Africa found a two to one preference for the blood-based self-test [18]. Thus, future studies might explore different combinations of test choices, such as provider-administered oral tests and blood-based self-tests, to assess which tests are the most popular and what combination of testing choices should be available in order to maximize test uptake.

The OraQuick HIV self-test kit used in this study has slightly lower sensitivity (probability that someone HIV-infected tests HIV-positive = 91.7%), but higher specificity (probability that someone not HIV-infected tests HIV-negative = 99.9%) [19] compared to the SOC test (Colloidal Gold, sensitivity = 99.8% and specificity = 98.5% [20]). Thus, there is a slightly higher false negative rate for the self-test compared to the provider-administered SOC test but a slightly higher false positive rate for the SOC test. Both tests are rapid antibody-based tests and will not detect an HIV-infection during the first 23–90 days [21]. However, if testing rates and frequency increase when self-testing is available, the net impact will be the detection of more HIV-infections despite the slightly lower sensitivity.

There are a number of limitations to this study that should be considered in interpreting study findings. First, as previously mentioned, our text message was not very detailed and many receiving it may not have understood what it meant. This may have limited the number of participants who came to the clinics for the self-test and weakened the impact of the intervention. Second, our method of selection of eligible participants via the North Star Alliance health record system might have led us to include some who were not eligible and to exclude some who were eligible due to data errors in the system. The inclusion of duplicate phone numbers in the system as well as the missing HIV-testing data on 38 female sex workers who self-tested indicates a fairly high level of delayed data entry or error in the system. It is also possible that some participants in our sample had recently tested or been diagnosed with HIV in a non-North Star clinic prior to the study and should have been excluded or tested for HIV during follow-up at a non-North Star clinic and were therefore misclassified in our data. We did, in fact, find two participants who did not have an HIV-positive status indication in the health record system, but when they came to the clinic and were offered HIV testing, disclosed to clinic staff that they were HIV-positive. These errors should all be non-differential by study arm, on average, and bias our results toward the null. Furthermore, the self-test kits were only available at clinics located in Kenya, which may have hampered access for some who traveled outside of Kenya. However, prior to randomization, the clinic most recently visited was located in Kenya for all participants (Table 1), so this seems unlikely to have resulted in many missed opportunities. Thus, it is possible that the association we found is an underestimation of the true effect. It is possible that the increased testing rate among those in the intervention is partly due to curiosity about a new HIV test and over time the differences in testing rates may decline after self-testing becomes widely available. Longer follow-up studies are needed to assess the long-term impact of self-testing on testing rates and test frequency. Finally, our results cannot be generalized to all female sex workers in Kenya, let alone outside of Kenya, as we selected a sample meeting specific criteria and from a clinic-based health record system. Those who do not access healthcare at all may differ in important ways that impact HIV testing.

## Conclusions

We found that sending text messages announcing the availability of HIV self-test kits at North Star Alliance clinics in Kenya increased HIV testing rates among a sample of female sex workers who were inconsistent HIV testers. However, a number of issues need to be considered in designing clinic-based programs that include self-testing. First and foremost, oral self-testing should be thought of as complementary to existing services rather than replacing them. Many of the participants in our study chose the existing SOC HIV test when given a choice, but for others, self-testing was an attractive alternative, in that more people came to the clinic to test when learning about the availability of self-test kits. Thus, testing choices may be the key to maximizing HIV testing rates. Implementing an HIV self-testing program not only requires HTC counselor training and consideration of how self-testing fits into the current service model (e.g., costs and pricing, dissemination venues), but also design of appropriate information and counseling resources for clients. As we learned in this study, questions do arise during the testing process, so clients need to have some way to have their questions answered. For those with smart phones, the online video [14] may help. Offering or even requiring supervision the first time someone self-tests might be another option to ensure that people get their questions answered and feel confident in their ability to self-test in the future. Most of those who self-tested in our study chose to do so in the clinic with supervision even though it was not required, suggesting this is an acceptable option for many as they learn to self-test. Similarly, mechanisms for pre- and post-test counseling need to be established. Mandatory pre-test counseling might be combined with picking up self-test kits at a local clinic or pharmacy, but post-test counseling is more difficult to deliver because it depends on the client to seek out this service. In our study, about a quarter of participants who self-tested at home did not contact the HTC counselor for post-test counselling. Given the challenge of linkage to care for those who test in a clinic setting, some thought will be needed to design self-testing programs that facilitate linkage to care. Thus, policy makers and program implementers have a number of challenges to address before self-testing is rolled-out in clinic systems such as the North Star Alliance. Roll-out should be coupled with evaluation of the various policy and programmatic decisions in an effort to maximize the impact self-testing has on controlling the HIV epidemic.
